# Assessment the value of Pyroptosis-Associated Gasdermin family genes in hepatocellular carcinoma: A Multi-Omics Comprehensive Analysis

**DOI:** 10.7150/jca.88887

**Published:** 2024-02-12

**Authors:** Changhong Wei, Jiamin Zhou, Wenfu Tao, Lixian Qin, Keke Zhang, Jieshan Huang, Ling Gao, Sufang Zhou

**Affiliations:** 1 Department of Clinical Laboratory, The Fifth Affiliated Hospital of Guangxi Medical University, Nanning, China.; 2 Department of Clinical Laboratory, The First People's Hospital of Nanning, Nanning, China.; 3 Department of Biochemistry and Molecular Biology, School of Basic Medicine, Guangxi Medical University, Nanning, China.; 4 Key Laboratory of Biological Molecular Medicine Research (Guangxi Medical University), Education Department of Guangxi Zhuang Autonomous Region, Nanning, China.

**Keywords:** Hepatocellular carcinoma, GSDMs, immune infiltration, Bioinformatics analysis, GSDME, Prognosis

## Abstract

**Background:** Hepatocellular carcinoma (HCC) is one of the common primary cancers of the liver worldwide and leading cause of mortality. Gasdermins (GSDMs) family genes play an important role in the regulation of the normal physiological processes and have been implicated in multiple diseases. However, little is known about the relationship between different GSDMs proteins and HCC. The aim of this study was to explore the potential relationship between the expression, prognosis, genetic variation and immune infiltration of GSDMs family genes and HCC.

**Methods:** We used different bioinformatics common public databases such as GSCA, GEPIA, UALCAN, HPA, Kaplan-Meier Plotter, LinkedOmics, GeneMANIA, STRING, cBioPortal, TIMER and TISIDB to analyze the differential expression of the different GSDMs, prognostic value, genetic alterations, immune cell infiltration and their functional networks in HCC patients.

**Results:** All the members of the GSDMs family exhibited elevated mRNA expression levels in LIHC compared to the normal tissues, while only GSDMB, GSDMD and GSDME showed enhanced protein expression. The mRNA expression of most GSDMs members was found to be elevated in HCC patients at stages I-III (clinical stage) compared to the normal subjects. The expression of GSDMD was correlated with OS and DSS of patients, whereas GSDME was correlated with OS, DSS and RFS of patients. Gene amplification was observed to be main mode of variation in members of the GSDMs family. KEGG pathway analysis showed that genes associated with different members of the GSDMs family were enriched in the pathways of *S*. *aureus* infection, intestinal immunity, ribosome and protein assembly, oxidative phosphorylation, osteoclast differentiation and Fc gamma (γ) R-mediated phagocytosis. In addition, expression of both GSDMA and GSDME were found to be correlated most significantly with infiltration of immune cells, while GSDMA and GSDME somatic cell copy number alteration (CAN) were correlated significantly with the infiltration of immune cells. All GSDMs were noted to be associated with distinct subtypes of immune cells, except GSDMC.

**Conclusions:** Our findings have provided useful insights to better understand the roles and functions of GSDMs in HCC that can provide novel direction for developing therapeutic modalities for HCC, including immunotherapy.

## Introduction

Liver cancer remains a global health challenge, and the incidence of this dreaded disease has increased significantly in many countries in recent years 1. As the main histological type of the liver cancer, hepatocellular carcinoma (HCC) accounts for approximately 90% of all the primary liver cancer cases^2^.The main risk factors for HCC, including hepatitis B and C virus infection, alcohol intake and aflatoxin B1 ingestion, among others are well known. Although the treatment options for HCC patients have markedly improved in the past decades, the clinical prognosis of patients have remained poor, with an overall survival rate (OS) of less than 30% at 5 years after resection for intermediate to advanced HCC^3^. Therefore, in addition to identification of novel biomarkers that can be used for the therapeutic stratification, it is important to search for more sensitive biomarkers that can be effectively used to determine the diagnosis, prognosis and progression of HCC. 4.

Gasdermins (GSDMs) are a recently discovered family of proteins located on the four different chromosomes, consisting of GSDMA, GSDMB, GSDMC, GSDMD, GSDME (also known as DFNA5) and DFNB59 (also termed as pejvakin) 5, 6. They can play an important role in the regulation of normal physiological processes and in a variety of diseases, such as skin diseases, asthma, hearing loss and cancer. 7, 8. However, only recently, several members of the GDSMs family have been found to alter the plasma membrane permeability during the different forms of regulated death, and have attracted significant interest for their role in both inflammation and host defense 9. GSDM pores can effectively disrupt the integrity of the cell membranes and trigger cell death by releasing their cellular contents including the various inflammatory cytokines outside of the cell. This process has been referred to as GSDM-mediated cell death as pyroptosis 10, 11. In addition, increasing evidences have suggested that GSDMS can also inhibit or promote infection and cancer, thereby implying a complex link between GSDMS and the onset and development of inflammation and pyroptosis 12-14. The functional differences between the GSDMs family proteins have also been questioned.

However, the diagnostic, prognostic value and molecular mechanisms of the GSDMs in HCC remain unclear. The main objective of this study was to explore this association by collecting the data from a series of public databases and performing bioinformatics analysis to further determine the potential role of GSDMs gene family members, which can provide sufficient scientific evidence for the prognosis and treatment of HCC.

## Materials and methods

### GSCA Database Analysis

We have used the GSCA database (http://bioinfo.life.hust.edu.cn/GSCA/#/) to study the expression of GSDMs in 33 cancers. This is a comprehensive database employed for the genomic and immunogenomic cancer analysis^15^. GSCA integrates more than 10,000 multidimensional genomic data from TCGA for 33 cancer types and more than 750 small molecule drugs from GDSC and CTRP. Immunogenomics analysis was performed by using ImmuCellAI algorithm with 24 immune cells. In this study, we have used this database to perform a pan-cancer analysis of the family genes (comparison of cancerous and normal tissues).

### Gene Expression Profiling Interactive Analysis (GEPIA)

GEPIA2 (http://gepia.cancer-pku.cn/index.html) is an updated version of Gene Expression Profiling Interactive Analysis (GEPIA)^16^. It can integrate a large amount of the data from The Cancer Genome Atlas (TCGA) and Genotype-Tissue Expression (GTEx) projects 17. In our study, we have used this database to assess the possible differences in the gene expression between LIHC and normal tissues and to generate the scatter and box plots. The correlation between GSDMs and clinical staging was also evaluated using the statistical method of Pearson correlation coefficient.

### UALCAN Database Analysis

UALCAN (http://ualcan.path.uab.edu) is a comprehensive, integrated web-based resource, which provides access to a wide range of gene expression and the patient clinical data from the TCGA database and it is used for the differential gene expression, survival analysis, methylation analysis, and more. 18. In addition, it can facilitate comparison of the different subgroups of genes for the differential expression level analysis. In our study, we have used UALCAN to further validate the expression levels of GSDMs family genes, the results of their protein expression levels and their relationship with the tumor staging.

### Human protein atlas (HPA) Database Analysis

The HPA (https://www.proteinatlas.org/) database applies proteomics technologies to provide the different protein profiles, including the tissue profiles, cellular profiles and pathological profiles 19. We have applied this database to analyze the protein expression of GSDMs family genes in LIHC.

### Kaplan-Meier Plotter Database Analysis

Kaplan-Meier plotter (http://kmplot.com) is an online database that can be used to estimate the tumor survival prognosis for more than 50,000 genes in 21 different cancer types 20, 21. Based on the expression level of GSDMs, the LIHC samples were divided into two distinct groups to analyze their overall survival (OS), progression-free survival (PFS), relapse-free survival (RFS), disease-specific survival (DSS).

### cBioPortal Database Analysis

cBioPortal (http://cbioportal.org) is an open and intuitive web-based database for analyzing the multidimensional data from the various cancers and combining the genetic variants, clinical data and visualizations 22, 23. We have used this database to explore the potential variations in GSDMs in LIHC, including amplification, mutation and copy number variation, and to correlate them with the prognosis.

### String Database

STRING (https://string-db.org/) is a comprehensive and objective website on the protein interactions 24. We have performed a PPI network analysis through STRING to collect and integrate the differentially expressed GSDMs and their potential interactions.

### GeneMANIA Database

GeneMANIA (https://genemania.org/) is a server for exploring the various gene associations and gene interactions, which can aid to analyze the interactions and functions between the submitted gene lists through a large amount of association data 25. We have used this database to identify the various genes associated with GSDMs and used GeneMANIA to explore their different functions.

### LinkedOmics Database

LinkedOmics (http://www.linkedomics.org/) is a publicly available multi-omics online database containing multi-omics and clinical data for 32 different cancers from TCGA^26^. We have used this database to screen the top 50 most relevant genes for each GSDMs family member, constructed their heat maps and volcano maps, and performed KEGG enrichment pathway analysis for these relevant genes.

### TIMER Database

The Tumor Immune Estimation Resource (TIMER) (https://cistrome.shinyapps.io/timer/) is a user-friendly tool for the systematic evaluation of the possible correlations between the genes and the different immune cell infiltrates, which provides a web interface to six major analysis modules 27. We have used it to explore the potential relationship between the gene expression, somatic cell copy number alteration (CNA) and immune infiltration.

### TISIDB Database

TISIDB (http://cis.hku.hk/TISIDB/) is another online database employed for the analysis of tumor-immune system interactions 28. It integrates multiple data types and allows users to explore the association of a specific gene with tumor-infiltrating lymphocytes. We have used it to analyze the relationship between gene expression and the different immune subtypes.

### Cell lines and Culture

Human liver cancer cell lines Hep-G2, SK-Hep-1 and normal liver cell line LO2 were purchased from the Cell Bank of the Type Culture Collection Center of the Chinese Academy of Sciences. The three cell lines were routinely cultured in DMEM (Gibco) medium supplemented with 10% (v/v) fetal calf serum (Gibco) and 1% (v/v) penicillin and streptomycin solution (MCE). All cell lines were cultured in a 37°C, 5% CO2 incubator and passaged using standard cell culture techniques.

### Western blot

Total protein was extracted in RIPA lysis buffer (Solarbio, Beijing, China) containing a phosphatase and protease inhibitor cocktail. After electrophoresis, denatured proteins were transferred to a 0.4µm polyvinylidene fluoride (PVDF) membrane at 300mA for 90 minutes. Subsequently, the membrane was incubated with primary and secondary antibodies, including anti-GSDME (1:1000; Abcam) and anti-GAPDH (1:2000; Abcam), and HRP secondary antibody (1:20,000; Proteintech Group, Inc). Finally, the protein signal was visualized using the Lanxiang imaging system.

## Results

### mRNA expression levels of GSDMs in human cancers

**Figure 1:**
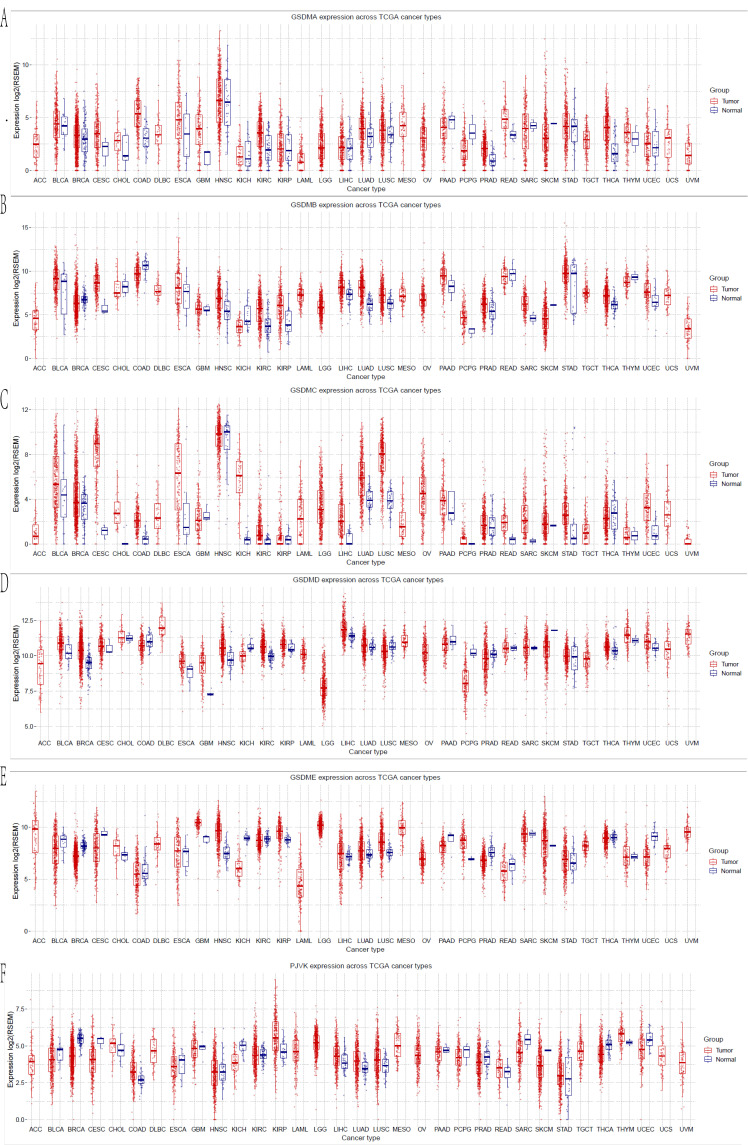
mRNA expression of GSDMs in the different cancers (GSCA).

The mRNA expression levels of each gene in the GSDM family were determined between the cancerous and normal tissues in pan-cancer using the GSCA database (**Figure 1**). Six different members of the GSDMs have been previously identified in humans. We noticed that all the GSDMs were differentially expressed in HCC tissues compared with the normal tissues. It was found that all of them were markedly elevated in the cancerous tissues compared with normal tissues, especially GSDMC and GSDMD were significantly upregulated with statistically significant differences (p<0.05), while the other members (GSDMA, GSDMB, GSDME and PJVK) were only elevated in the cancerous tissues with no statistically significant differences (p>0.05) (**Table 1**).

### mRNA and protein expression levels of GSDMs in LIHC

**Figure 2:**
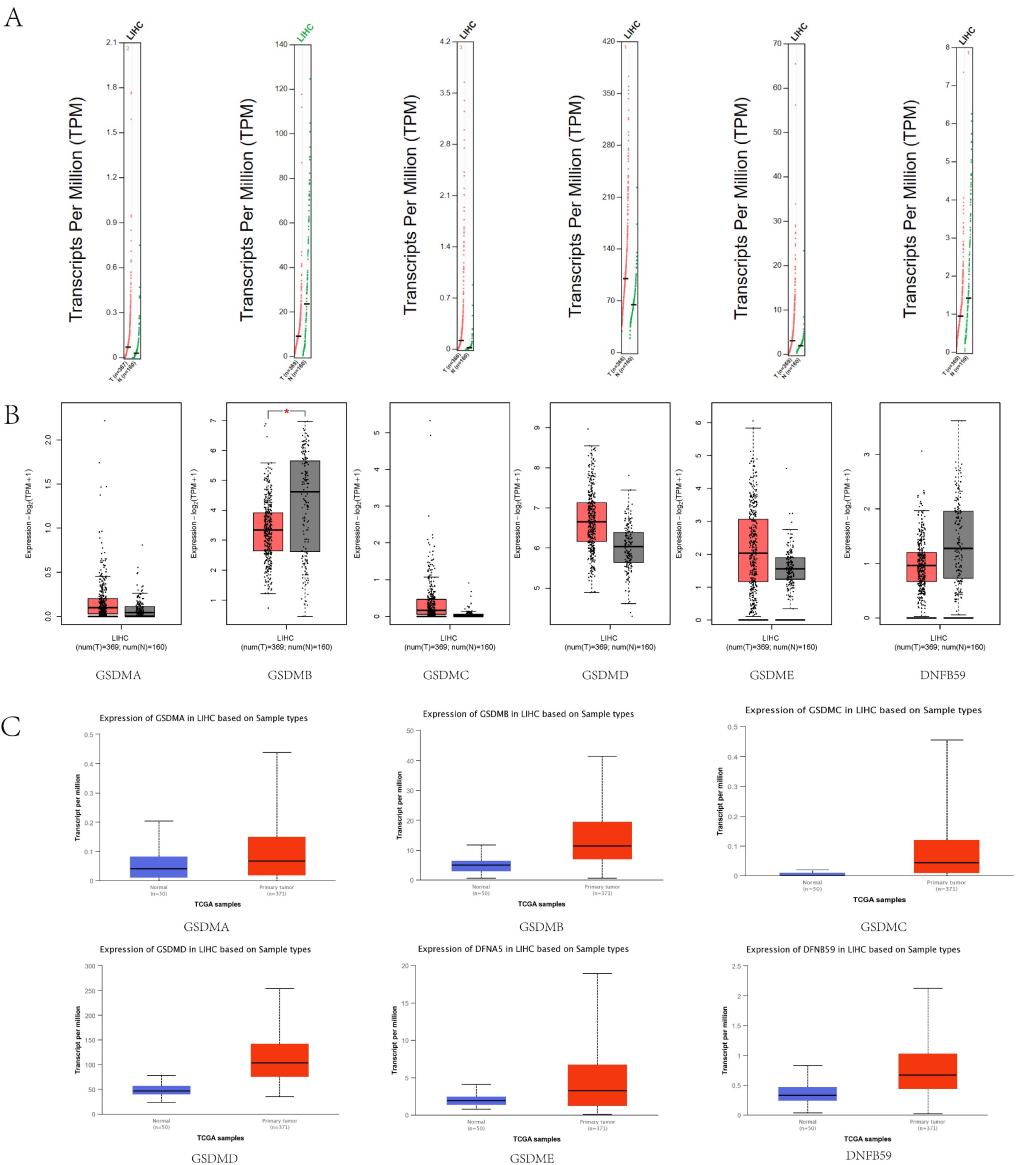
mRNA expression levels of GSDMs in LIHC. A: Scatter diagram (GEPIA), B: Box plot (GEPIA), C: Box plot (UALCAN).

The GEPIA database was used to analyze the mRNA expression levels of GSDMs in LIHC (**Figure 2A, B**), including in 369 LIHC tissues and 160 paraneoplastic tissues. The expression levels of GSDMB and DFNB59 were observed to be significantly higher in paraneoplastic tissues than in the cancerous tissues, and the expression of other GSDMs family members (GSDMA, GSDMC, GSDMD and GSDME) was higher in the cancerous tissues than paraneoplastic tissues. To further verify the accuracy, we also examined the mRNA expression levels of GSDMs in LIHC using the UALCAN database (**Figure 2C**), and found that the expression levels of all GSDM family members were higher in HCC tissues than in normal tissues.

**Figure 3:**
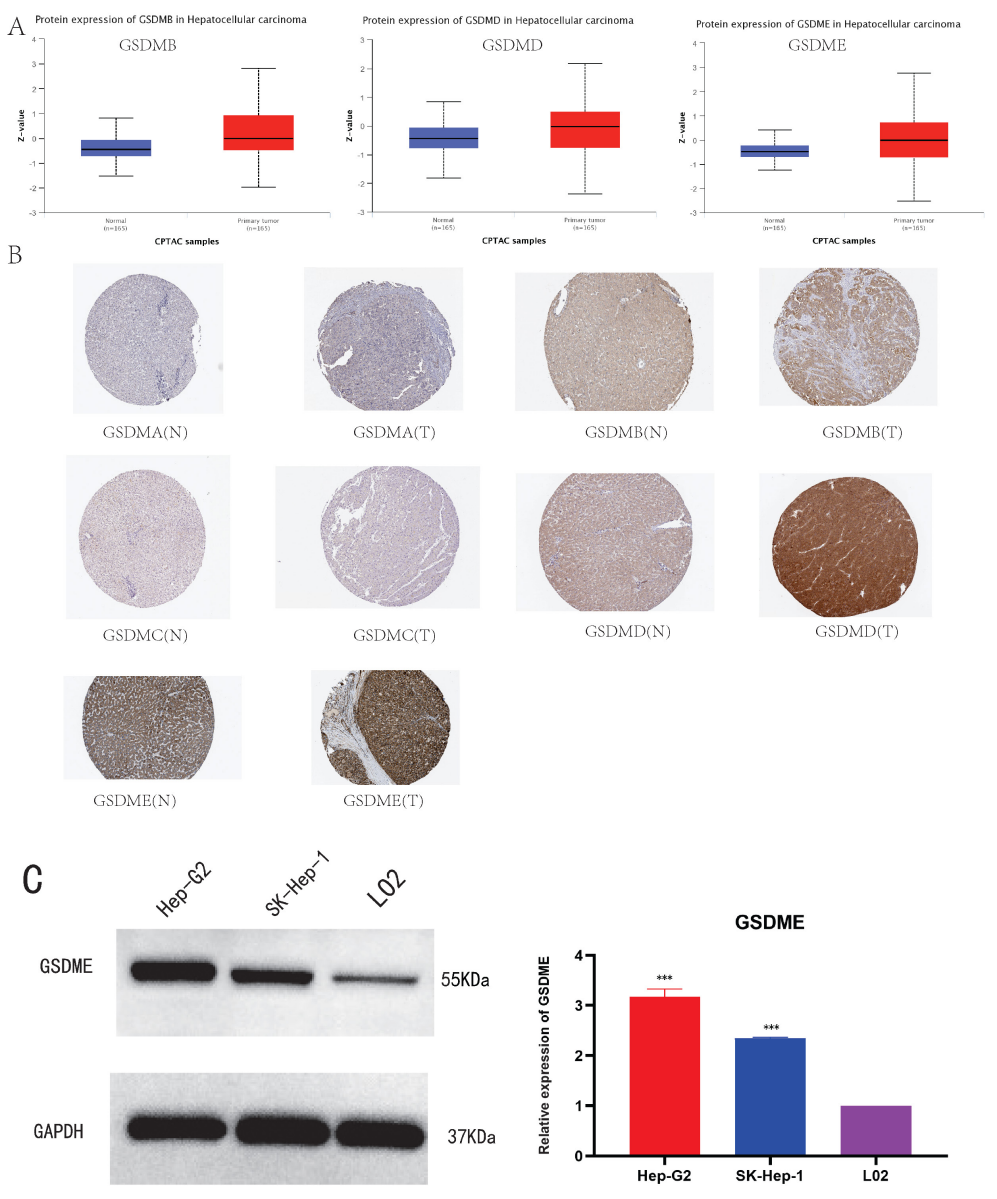
The protein expression levels of GSDMs in LIHC (A: UALCAN, B: HPA, C: GSDME protein levels in 2 LIHC cell lines and human normal liver cell. * P<0.05, **P<0.01, ***P<0.001).

We analyzed the expression of GSDMs proteins in HCC tissues and their normal tissues using the UALCAN and HPA databases for the comparison. It was noted that except for the missing information of GSDMA, GSDMC and DFNB59, the protein levels of GSDMB, GSDMD and GSDME were substantially increased in LIHC tissues compared to the normal tissues (**Figure 3A**). We used the HPA database to analyze the immunohistochemical staining results of Gasdermins protein in liver cancer tissues and normal liver tissues, the protein levels of GSDMB, GSDMD and GSDME staining concentrations were found to be increased in LIHC tissues compared with normal tissues, while GSDMA, GSDMC levels did not change (**Figure 3B**).Based on the above bioinformatics analysis, we further evaluated the expression of GSDME protein in LIHC, and Western blot detected GSDME in LO2 cell line (normal liver cells) and 2 human liver cancer cell lines (Hep-G2 and SK-Hep-1). The expression of GSDME was significantly increased in liver cancer cell lines (**Figure 3C**), which confirmed the results of the above bioinformatics analysis.

### Relationship between the expression of GSDMs and the clinical features

**Figure 4:**
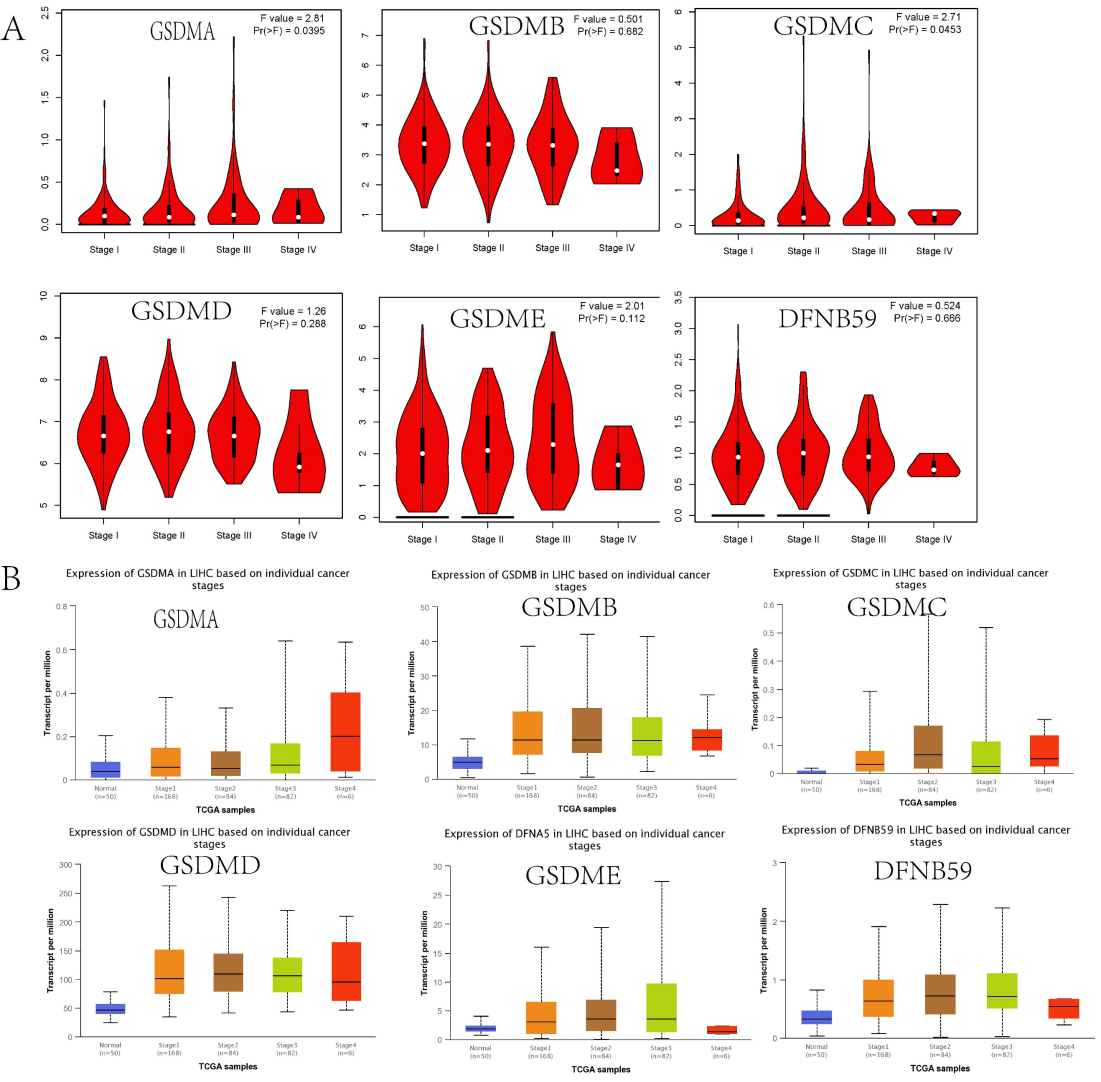
Correlation between GSDMs expression and the tumor stage in HCC patients (A: GEPIA, B: UALCAN).

We first examined the relationship between HCC tumor stage and GSDMs using the GEPIA database. The results indicated that there was a significant difference between the GSDMA and GSDMC groups (p<0.05), while there was no significant difference found between the other groups (**Figure 4A**). Moreover, to verify this finding, we analyzed the relationship between LIHC tumor stage and GSDMs expression using the UALCAN database. We found that the expression of GSDMs was significantly higher with higher tumor stage, which was particularly evident in GSDMB, GSDMD, and DFNB59 (**Figure 4B**).

**Figure 5:**
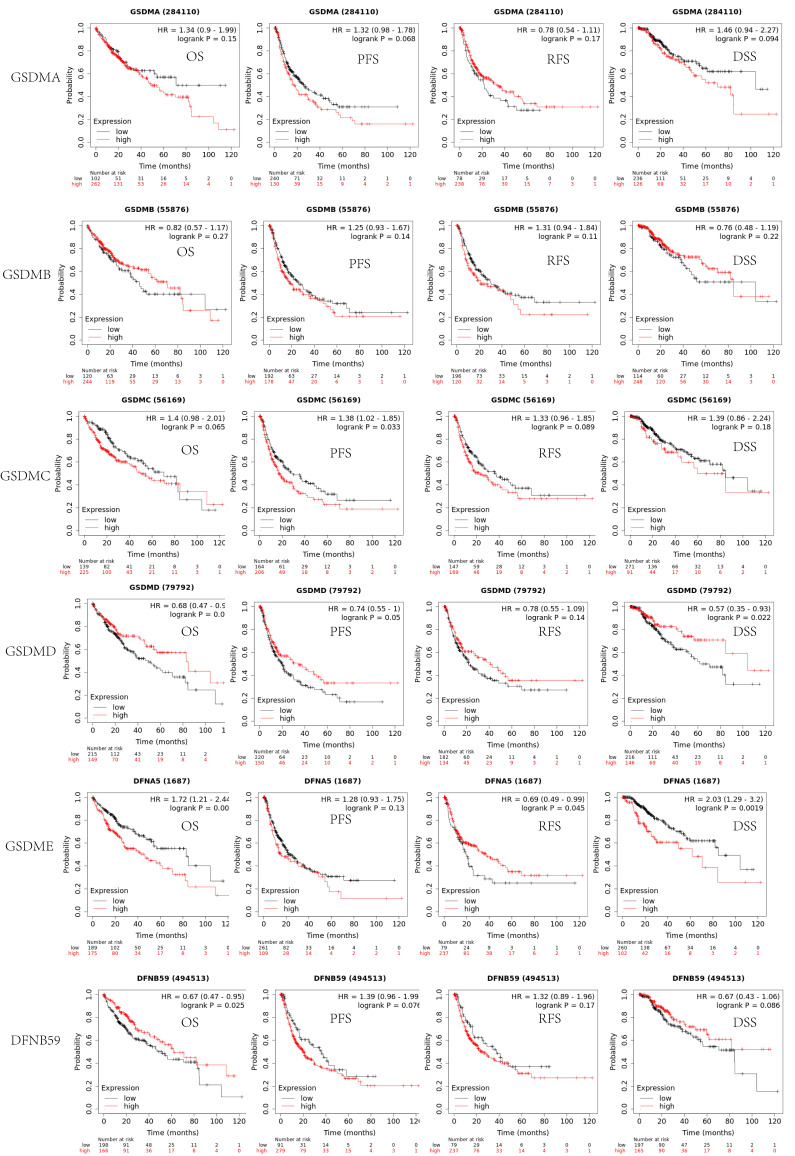
The survival analysis of the patients of LIHC by analyzing the expression levels of GSDMs (Kaplan-Meier plotter).

In addition to determining the tumor staging, we also evaluated the effect of GSDMs on the prognostic value of LIHC using the Kaplan-Meier Plotter database. As shown in Figure 5, the expression levels of both GSDMA and GSDMB did not significantly affect the prognosis (OS, PFS, RFS and DSS) of LIHC (p>0.05). The patients in the GSDMC low expression group exhibited better PFS than the high expression (p<0.05). The patients in the GSDMD high expression group showed better OS and DSS than the low expression group (p<0.05). The patients in the GSDME low expression patients in the GSDMD high expression group displayed better OS and DSS than the high expression group (P<0.05), while patients in the GSDME high expression group had better RFS than the low expression group (P<0.05). Moreover, the OS of patients in the high expression group of DFNB59 was substantially better than that in the low expression group (P<0.05), but no significant differences were observed in other groups (**Figure 5**).

### Analysis of genetic alterations and prognosis of GSDMs

**Figure 6:**
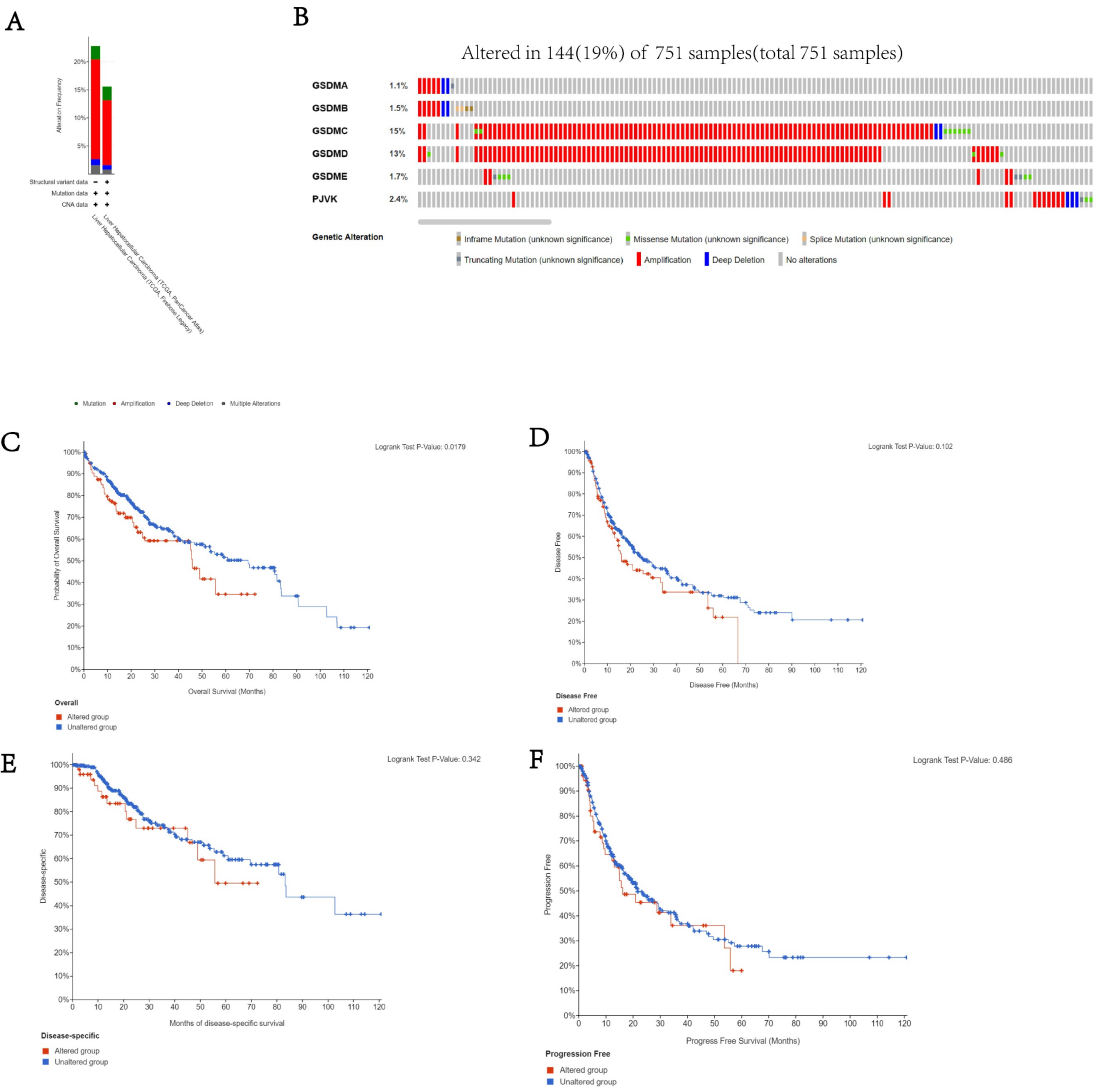
Genetic alterations related to the GSDMs in LIHC. The total frequency of GSDMs alterations (A). Details of the genetic alterations of each member of GSDMs in each sample of LIHC (B). K-M comparing OS of patients with and without GSDMs alterations (C). KM plot comparing DFS in the patients with and without GSDMs alterations (D). KM comparing DSS in patients with and without GSDMs alterations (E). KM comparing PFS in the patients with and without GSDMs alterations (F).

We used the cBioPortal database to study the genetic variation among the different members of the GSDM family. It was found that 19% (144/751) of patients had genetic variations and amplification was the most common mutation among the GSDMs isoforms (**Figure 6A**). In addition, GSDMA, GSDMB, GSDMC, GSDMD, GSDME and DFNB59 were respectively altered in 1.1, 1.5, 15, 13, 1.7 and 2.4% of HCC specimens (**Figure 6B**). In addition, we examined the relationship between the genetic alterations in the GSDMs and the prognosis of HCC patients (OS, DFS, PFS and DSS), and found from Kaplan-Meier plots as well as log-rank tests that the various genetic alterations in the GSDMs were associated with shorter OS (p<0.05) (**Figure 6C-F**).

### Gene-gene, protein-protein interaction analysis of GSDMs family members

**Figure 7:**
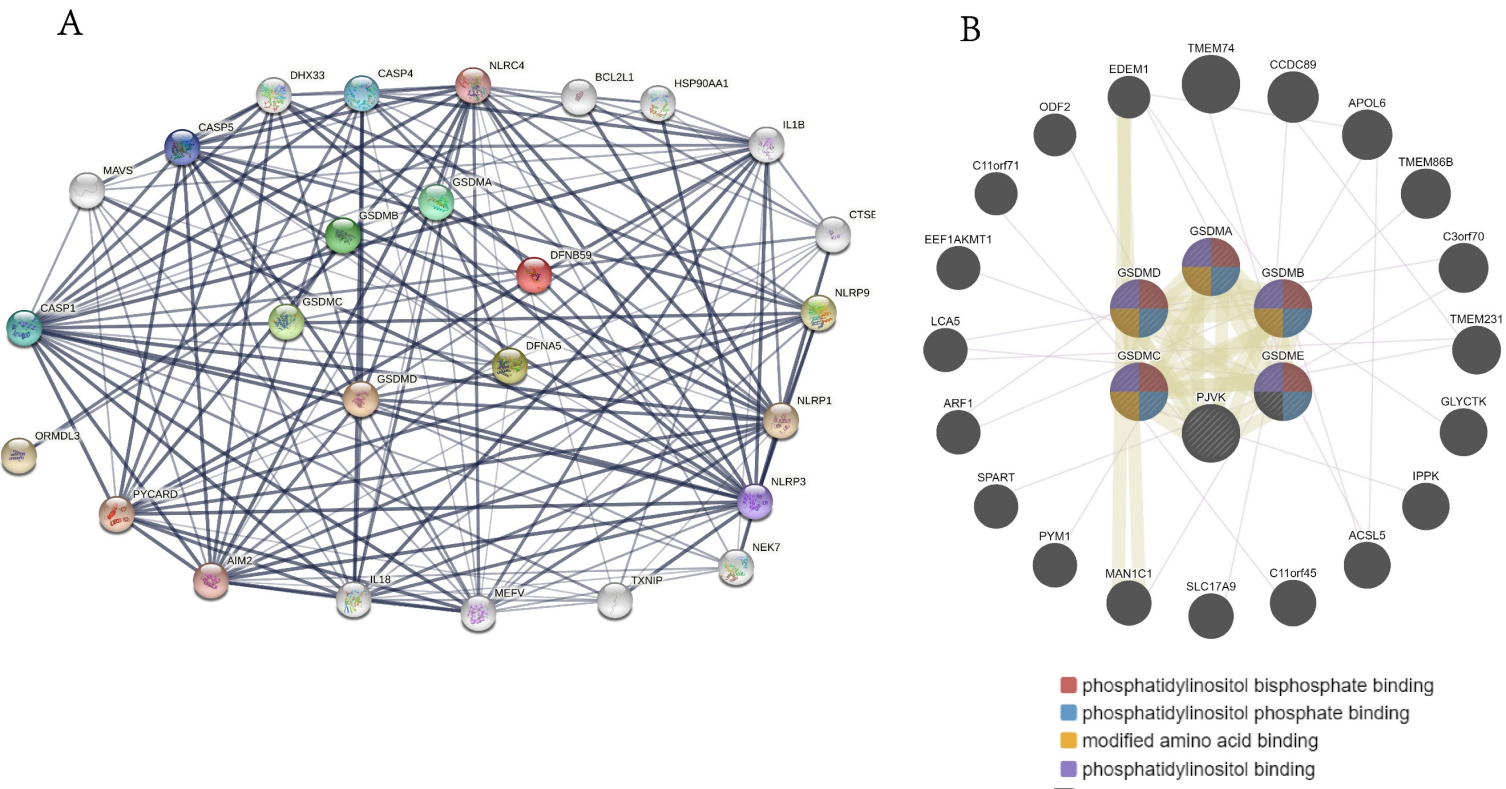
Interaction network diagram of GSDMs family genes and the proteins in LIHC. (A) Interaction network map between proteins encoded by GSDMs (STRING). (B) PPI network of GSDMs (GeneMANIA).

We performed protein-protein interaction PPI network analysis on GSDM family members using STRING database to explore their potential interactions. Ultimately, we obtained the protein interaction networks including 26 distinct nodes and 156 edges (**Figure 7A**), and these proteins were shown to be mainly associated with the signaling pathways regulating apoptosis and pyroptosis. We also used the GeneMANIA database to identify the various genes associated with GSDMs and found that 20 main related molecules (e.g. TMEM74, CCDC89, APOL6, TMEM86B, C3orf70, TMEM231 and GLYCTK) acted in combination with them. The potential functions of these genes were mainly related to maintenance of the cellular morphology, metabolic regulation, signaling and various physiological functions of phosphatidylinositol in the cells (**Figure 7B**).

### Co-expression network of GSDMs and potential functions in HCC

**Figure 8:**
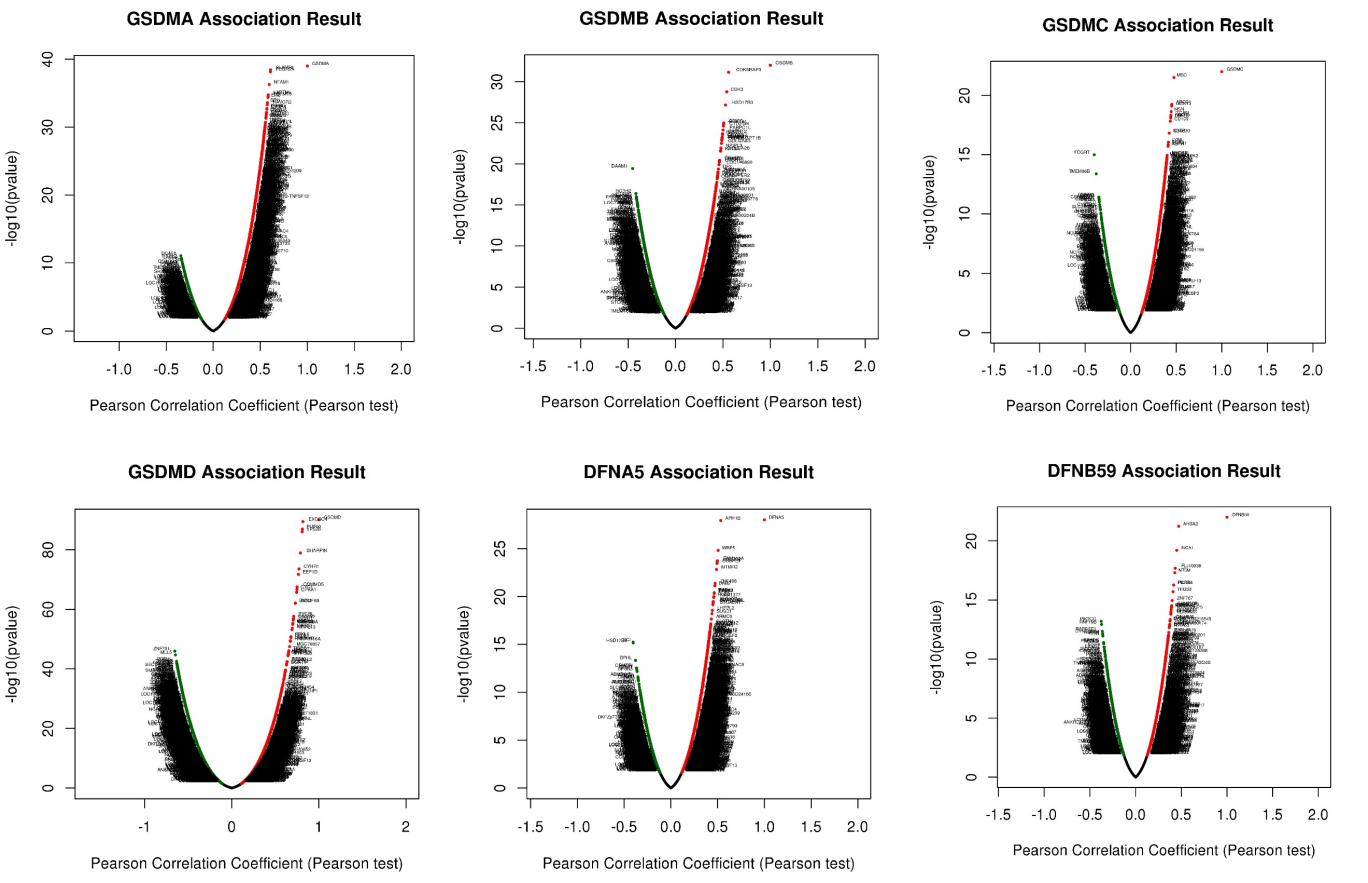
Volcano map of the top 50 genes co-expressed with GSDMs (LinkedOmics).

**Figure 9:**
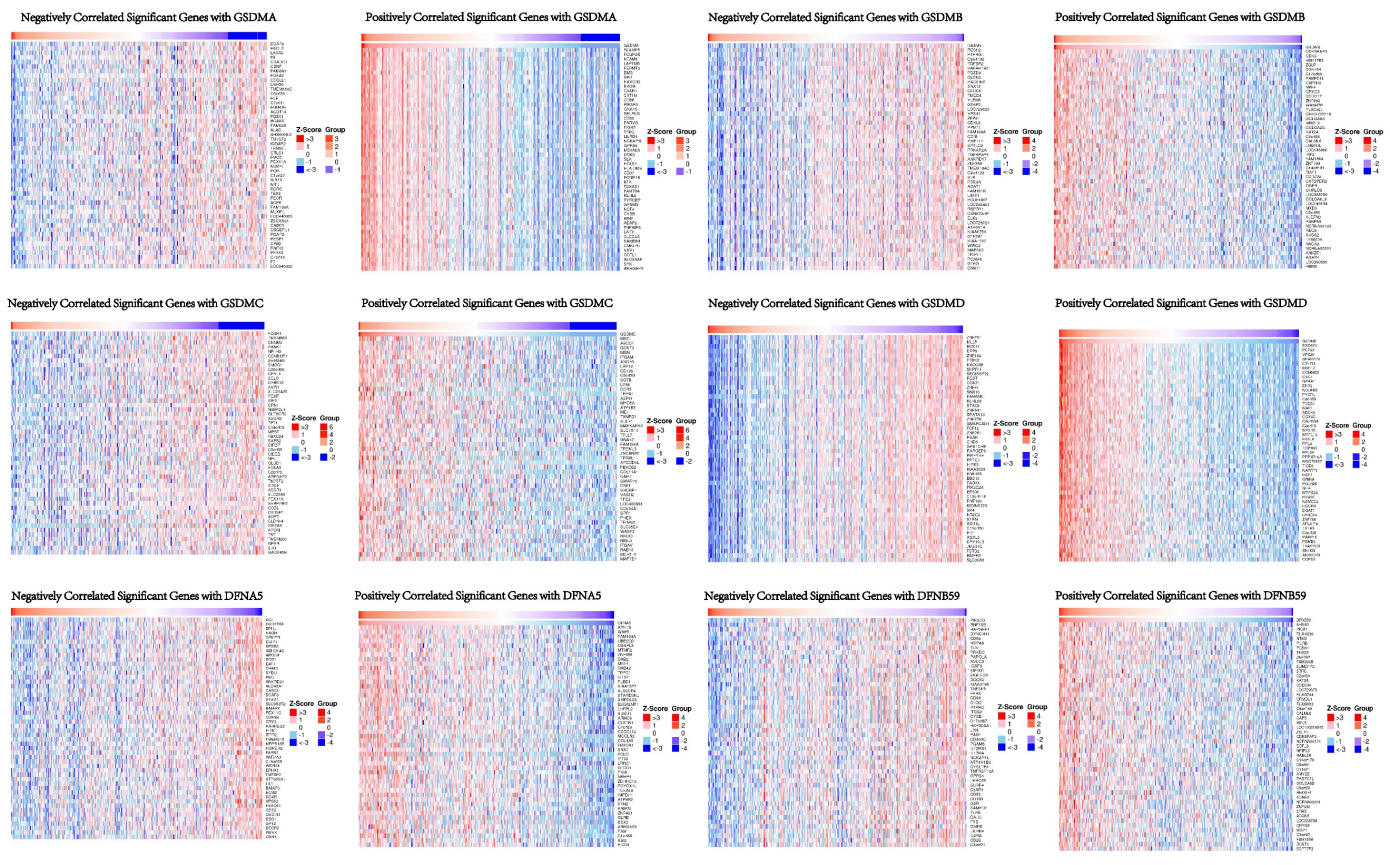
Heat map of the top 50 genes co-expressed with GSDMs in LIHC (LinkedOmics).

In order to understand the biological significance of GSDMs in HCC, we analyzed the co-expression network of this family of genes in the LIHC cohort using LinkedOmics. The correlation between GSDMs and the differentially expressed genes in LIHC has been presented as a volcano plot (**Figure 8**), with positively correlated genes in red and negatively correlated genes in blue. The heat map showed both the positive and negative correlations of the top 50 genes in LIHC with the GSDMs (**Figure 9**). The expression of GSDMA was found to be strongly positively correlated with the expression of SLAMF8, FCGR2A and NFAM1, while it was strongly negatively correlated with DCAF8, HSDL2 and LASS2. On the contrary, the expression of GSDMB was noted to be strongly positively correlated with the expression of CDK5RAP3, CDK3 and HSD17B3, and strongly negatively correlated with DAAM1, RC3H2 and PTPRG. In addition, the expression of GSDMC was observed to be strongly positively correlated with the expression of MSC, ABCC1 and GCNT3, and strongly negatively correlated with FCGRT, TMEM86B and CNNM3.

**Figure 10:**
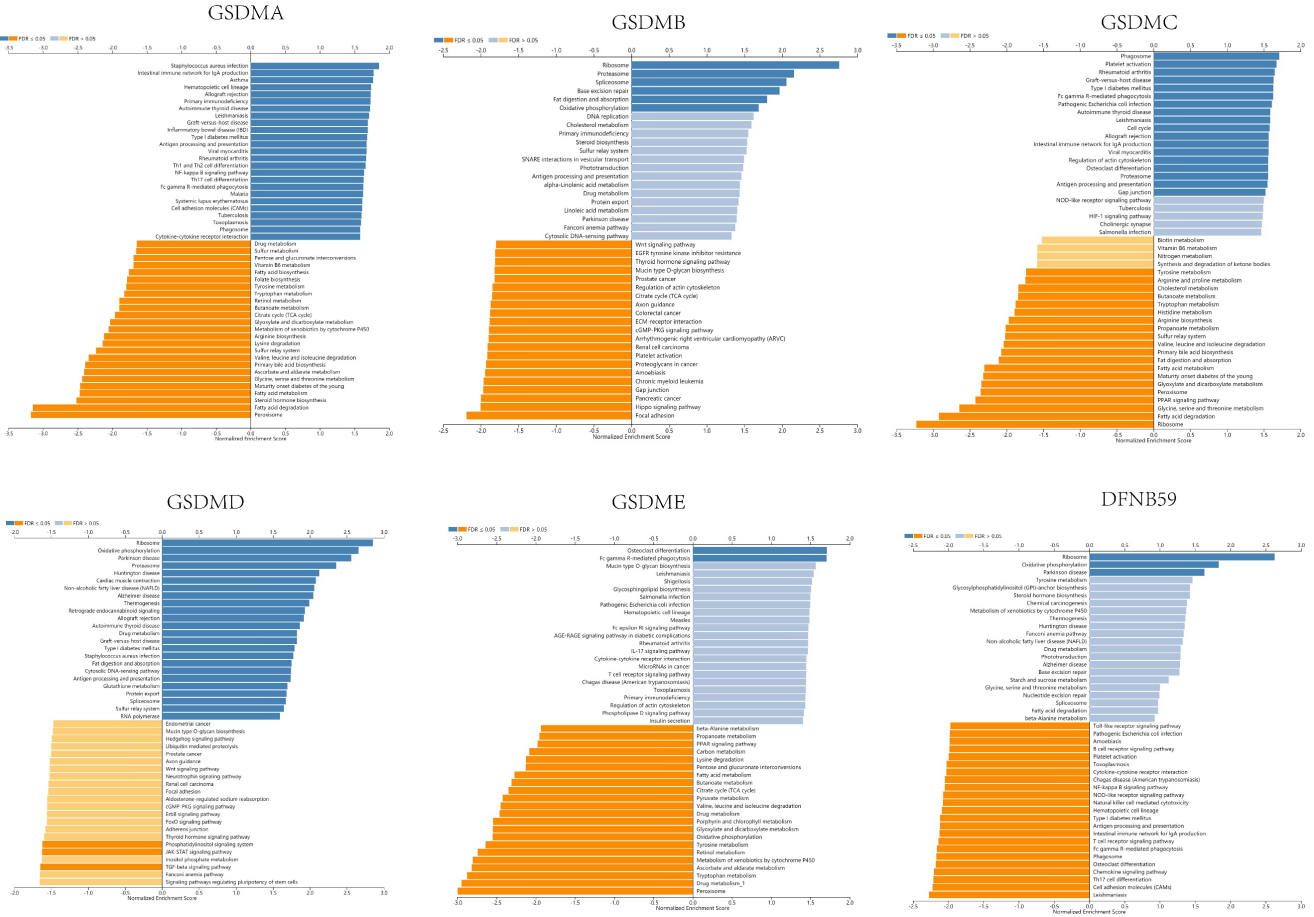
KEGG pathway analysis of the various genes associated with GSDMs in LIHC (LinkedOmics).

The expression of GSDMD was strongly positively correlated with EXOSC4, PUF60 and UPS28, and strongly negatively correlated with ZNF791, MLL5 and BoD1L. The expression of GSDME was found to be strongly positively correlated with the expression of APH1B, WBP5 and FAM164A, and strongly negatively correlated with DCI, HSD17B8 and BPHL. The expression of DFNB59 was observed to be strongly positively correlated with the expression of AHSA2, INCA1 and FLJ10038, and strongly correlated with PIK3CG, RNF19B and RHPGEF1 negative correlation. Kyoto Encyclopedia of Genes and Genomes (KEGG) pathway analysis indicated that co-expressed genes were mainly enriched in S. aureus infection, intestinal immunity, ribosome and protein assembly, oxidative phosphorylation, osteoclast differentiation, and Fc γ R-mediated phagocytosis pathways (**Figure 10**), thereby suggesting an effect on cell death and immune activation in HCC.

### Correlation between the expression of GSDMs and the level of immune infiltration in HCC tissues

**Figure 11:**
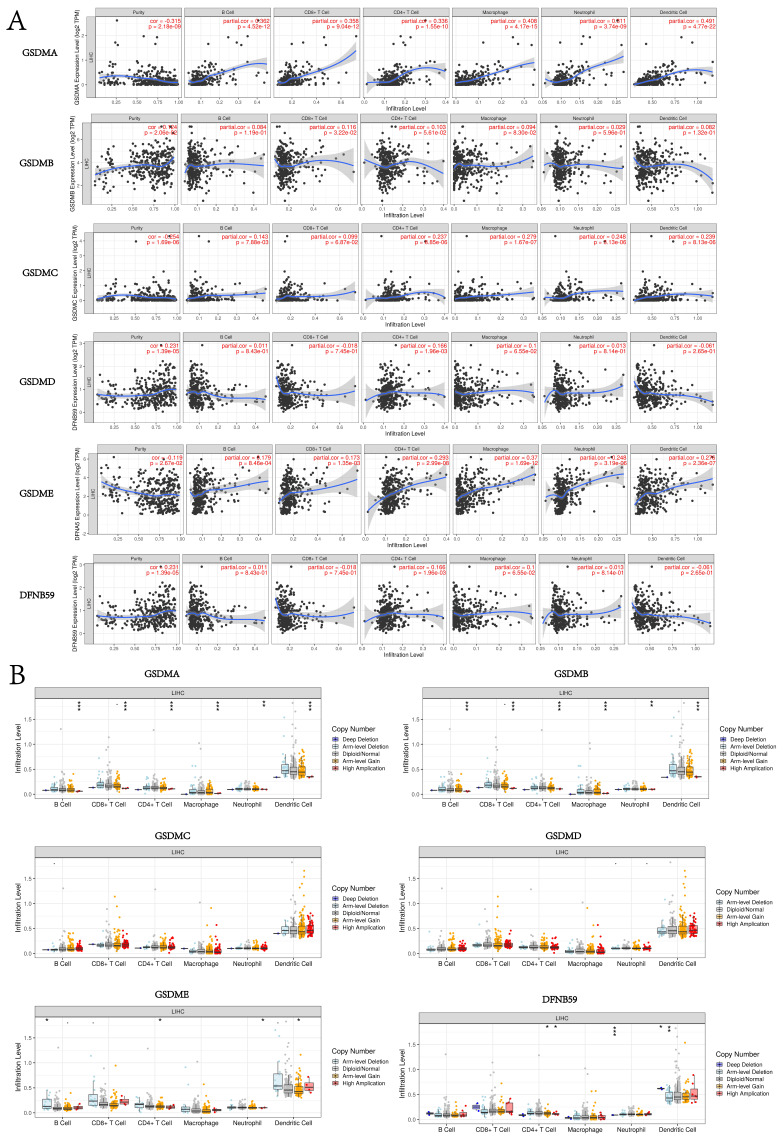
Relationship between GSDMs expression in LIHC and immune infiltration (TIMER). Correlation between the abundance of immune cells and the expression of GSDMs (A). Effect of CNV of GSDMs on the distribution of the various immune cells (B). (*p < 0.05; **p < 0.01; ***p < 0.001).

**Figure 12:**
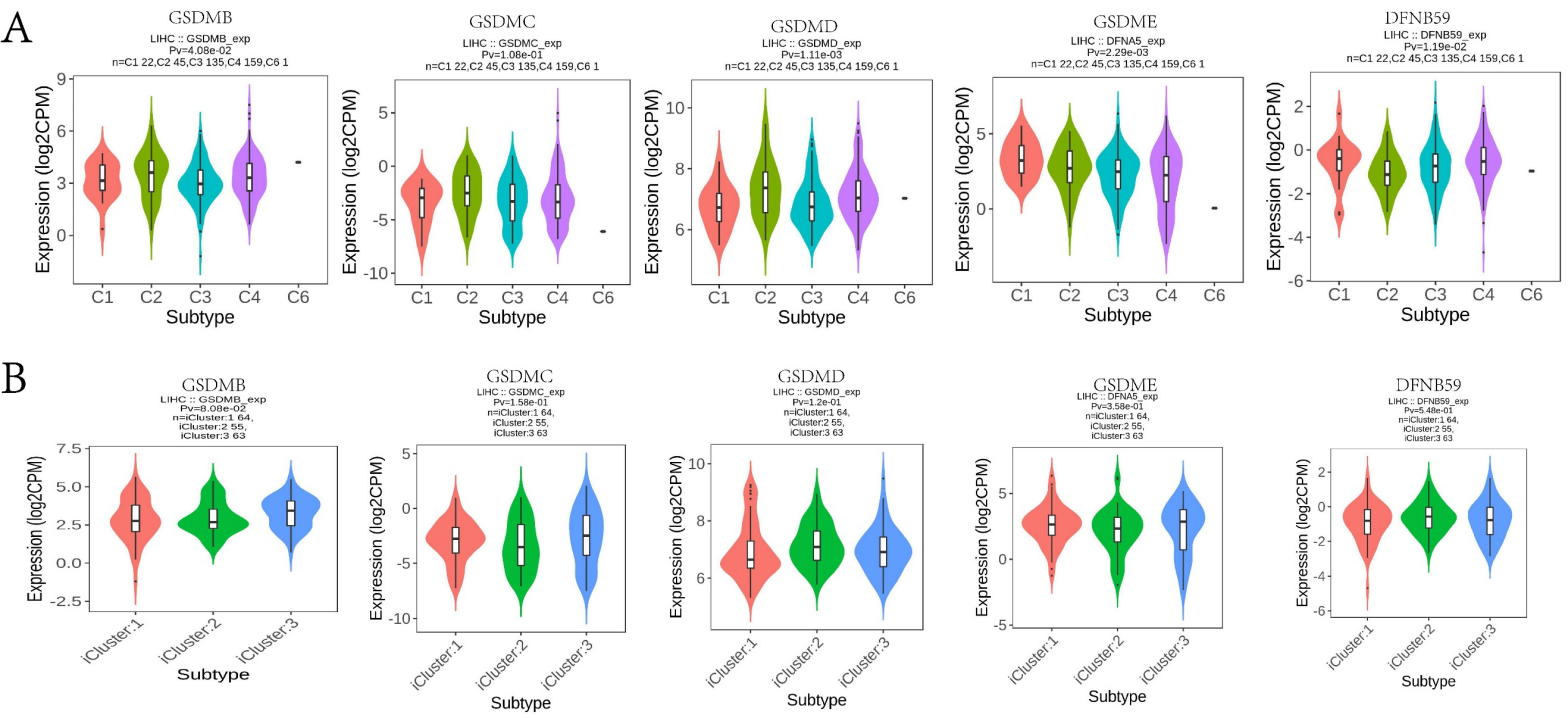
Correlation between the expression of GSDMs in LIHC and the level of immune infiltration. (A) Correlation between the expression of GSDMs in LIHC and immune subtypes (C1: Wound healing; C2: IFN-γ dominant; C3: inflammatory; C4: lymphocyte depleted; C5: immunologically quiet; C6: TGF- β dominant). (B) Association of GSDMs expression with different molecular subtypes of HCC (TISIDB).

In this study, the TIMER database was used to explore the potential correlation between GSDM members and immune cell infiltration. GSDMA expression showed a significant negative correlation with the tumor purity of LIHC (p < 0.05) and a significant positive correlation with the degree of infiltration of B cells, CD8+ T cells, CD4+ T cells, macrophages, neutrophils, and dendritic cells. The expression of GSDMB was significantly and positively correlated with the tumor purity of LIHC as well as the degree of infiltration of CD8+ T cells and macrophages. The expression of GSDMC was significantly and negatively correlated with the tumor purity of LIHC (p < 0.05), while it was significantly and positively correlated with the degree of infiltration of B cells, CD4+ T cells, macrophages, neutrophils and dendritic cells. The expression of GSDMD was significantly and positively correlated with the GSDMD expression was significantly and positively correlated with the tumor purity of LIHC and the degree of infiltration of CD4+ T cells. GSDME expression was significantly and negatively correlated with the tumor purity of LIHC (p < 0.05), while it was significantly and positively correlated with the degree of infiltration of B cells, CD8+ T cells, CD4+ T cells, macrophages, neutrophils and dendritic cells. DFNB59 expression was significantly and positively correlated with the tumor purity and the degree of infiltration of CD4+ T cells (**Figure 11A**). We also investigated the degree of immune cell infiltration of GSDMs in the tumors with different somatic cell copy number alterations (**Figure 11B**). It was found that the copy number variation (CNV) of GSDMA and GSDMB were significantly correlated with the degree of infiltration of B cells, CD8+ T cells, CD4+ T cells, macrophages, neutrophils and dendritic cells. However, the CNV of GSDME was significantly correlated with the degree of infiltration of B cells, CD4+ T cells, neutrophils and dendritic cells, and that of DFNB59 was significantly correlated with the degree of infiltration of CD4+ T cells, neutrophils and dendritic cells. In addition, the TISIDB database was used to explore the relationship between GSDMs and the various immune subtypes and the molecular subtypes of the tumors (**Figure 12**). We found that the expression levels of GSDMB, GSDMD, GSDME, and DFNB59 were significantly correlated with the immune subtypes of HCC (p < 0.05), except for the missing information of GSDMA, while none of the expression levels of GSDMs could be directly correlated with the molecular subtypes of HCC.

## Discussion

Currently, HCC remains the leading cause of cancer-related deaths in the world, seriously endangering the lives and health of world population 29. There are six family genes of GSDMs in humans: *GSDMA*, *GSDMB*, *GSDMC*, *GSDMD*, *GSDME* (*DFNA5*) and *DFNB59* (*PJVK*). Since the initial discovery of the GSDM family genes more than two decades ago, the diverse biological functions of the GSDMs family genes have been extensively investigated. At the beginning of the 21st century, GSDMs genes were first reported as candidates for causing alopecia-like skin mutations in mice^30^. Over the course of more than 15 years, the exact biological functions of these diverse proteins have gradually become known. In particular in recent years, these proteins have been identified to be closely linked to the regulation of cellular activity and inflammation^31^. The real breakthrough was the identification of GSDMD as a key executor of pyroptosis^11^, ^32^, a new form of the programmed death associated with inflammation, the main mechanism of which is the ability of *GSDM* genes to form distinct holes in the cell membrane and trigger cell death after their N-terminal activation. Therefore, most studies have focused on the site at which *GSDM* genes can be cleaved by the caspases or other enzymes, thereby activating specific *GSDM* genes to produce N-terminal and subsequently form pores in the cell membranes, thereby inducing cell death. However, so far, only *GSDMB*, *GSDMD* and *GSDME* have been only extensively studied in pyroptosis 9, but little is known about the specific functions of *GSDMA*, *GSDMC* and *DFNB59 (PJVK*) genes. In addition, the GSDMs have been associated with the regulation of various hallmarks of cancer, but whether they can effectively suppress or promote cancer remains controversial^33^. More importantly, the different roles of GSDMs family members in HCC progression remain to be elucidated. In this study, we have used the various public databases to reveal for the first time the aberrant expression of the GSDMs family as well as their relationship with the tumor staging, mutation, prognosis, and tumor immunity.

Here, we mainly found that the mRNA expression of all the six GSDM members were significantly increased in HCC tissues, thereby suggesting the possibility that they might play an important role as potential oncogenes in HCC. However, the expression levels of GSDMB and DFNB59 were found to vary in the different databases, probably due to the different number of the cases included in the studies included in the different databases. The consistency of GSDMs family expression trends implied that the relevant biological functions of GSDMs in HCC may be consistent. However, at the protein expression level (cancer tissues compared with the normal tissues), only the protein levels of GSDMB, GSDMD and GSDME were found to be highly expressed in HCC tissues, GSDMA and GSDMC levels largely remained unchanged, which may be caused by the fact that these two proteins have been less extensively studied.

Immediately after, we investigated the relation between the expression of these six GSDMs members with the clinical stage and prognosis of HCC, in order to analyze whether these family genes could function as oncogenes. We primarily found that only GSDMA and GSDMC were associated with the clinical stage (I-IV) in the GEPIA database, while the other GSDMs members were not associated with the clinical stage. However, in the UALCAN database, we observed that the mRNAs of these six GSDMs were highly expressed in the clinical stages (stages I-III) of HCC patients compared with the normal tissues, while the expression level of GSDME in stage 4 HCC patients was found to be the same as that of the normal tissues. This further established that GSDMs are primarily involved in the progression of HCC as oncogenes, but the advanced cancer tissues might interfere with the expression of GSDME. This point aroused our further interest in GSDME, and there have been several studies, which have indicated that GSDME were closely associated with the cancer development 34. For example, Triptolide inhibited head and neck cancer cell progression by inducing Gasdermin E (GSDME)-mediated cell pyroptosis 35, GSDME mediated lobaplatin-induced colorectal cancer cell pyroptosis downstream of ROS/JNK/Bax-mitochondrial apoptosis pathway and caused caspase-3/-9 activation 36. Moreover, GSDME can enhance the sensitivity of cisplatin to inhibit the progression of non-small cell lung cancer by triggering anti-tumor immune cell infiltration through promoting cellular pyroptosis^37^. Therefore, we believe that GSDME might have enormous potential to act as new tumor marker and biotherapeutic target for HCC.

In terms of clinical prognostic value, we found that GSDMC, GSDMD, GSDME and DFNB59 all displayed substantial prognostic value, but GSDMC was only associated with PFS, GSDMD was only associated with OS and DSS, and DFNB59 was only associated with OS in HCC patients, while GSDME was associated with both OS, RFS and DSS. These results suggested that GSDME might act as a potential biomarker for predicting the prognosis of liver cancer and a potential target for drug development.

It has been established that epigenetic alterations play a crucial role in development of the various malignancies 38. Interestingly, genetic analyses showed that the differentially expressed *GSDM* genes were frequently altered in HCC, predominantly by gene amplification, and patients with altered *GSDM* genes had a poorer OS prognosis, which might provide a theoretical basis for the tumor gene targeting therapy.

In addition, to analyze the functions of the GSDMs family, we performed data analysis using GeneMANIA, STRING and LinkedOmics. GeneMANIA and STRING results indicated that these proteins associated with GSDMs were mainly related to apoptosis, pyroptosis, phosphatidylinositol metabolism and cell signaling related pathways. KEGG pathway analysis using LinkedOmics constructs indicated that the various co-expressed genes were mainly enriched in *S. aureus* infection, intestinal immunity, ribosome and protein assembly, oxidative phosphorylation, osteoclast differentiation, and Fc γ R-mediated phagocytosis pathways, which provided strong evidence for the potential effects of GSDMs on the cell death and immune activation in HCC.

The tumor microenvironment plays a pivotal role in cancer development and immune infiltration is an important component of the tumor microenvironment that is highly relevant to the tumor diagnosis, progression, and prognosis^39^, ^40^. It has been reported that IL1β secretion usually requires proteolytic maturation of inflammasomes and membrane pore formation by gasdermin D (GSDMD) 41. GSDMD subcellular localization patterns have been found to be associated with CRC progression and immune response, and different subcellular locations of gasdermin D can predict the progression, immune microenvironment and prognosis of colorectal cancer^42^. In this study, TIMER and TISIDB databases were used to explore the possible correlation between the various members of GSDMs and immune cell infiltration. Our study showed that the expression of GSDMs directly correlated with the infiltration of six different immune cell types, and we particularly noted that both GSDMA and GSDME could be significantly correlated with the infiltration of immune cells. Among the correlation studies with various immune subtypes, GSDMD and GSDME showed the most significant correlation with immune subtypes. These findings suggested that GSDMs-mediated pyroptosis may play an important role in antitumor immunity by affecting immune cell infiltration, especially in GSDME.

## 5. Conclusions

In summary, we have demonstrated that the expression of GSDMs in LIHC was strongly correlated with the clinical features, prognosis and degree of immune cell infiltration. Thus, GSDMs family (especially GSDME) can serve as novel new biomarkers as well as potential therapeutic targets and can aid to improve the diagnosis and prognosis of LIHC. The results of these studies were based on the multidimensional bioinformatics analysis and cross-validated using the multiple databases, but a small number of results were inconsistent, and hence additional studies are needed to confirm these results.

## Figures and Tables

**Table 1:** Changes in the expression of GSDMs at the transcriptional level between HCC and the normal tissue (GSCA).

Cancer type	Gene symbol	Expression (tumor)	Expression (normal)	Fold change	P value	FDR	n_tumor	n_normal
LIHC	GSDMA	6.221008	5.688528	1.091988844	0.80559734	0.84726913	50	50
LIHC	GSDMB	233.328142	192.82729	1.209928061	0.22136767	0.31016007	50	50
LIHC	GSDMC	8.74009	0.562368	13.34619124	0.00022769	0.00095605	50	50
LIHC	GSDMD	4518.04776	2712.64172	1.665528174	0.0005572	0.00209117	50	50
LIHC	GSDME	236.633006	149.550688	1.581903893	0.09895109	0.16458745	50	50
LIHC	PJVK	19.11443	15.810798	1.207634589	0.118943	0.1909012	50	50
